# Hydrogen-Bonded Polymer Complex Thin Film of Poly(2-oxazoline) and Poly(acrylic acid)

**DOI:** 10.3390/polym9080363

**Published:** 2017-08-15

**Authors:** Chao Su, Jiaxing Sun, Xuejian Zhang, Duan Shen, Shuguang Yang

**Affiliations:** State Key Laboratory for Modification of Chemical Fibers and Polymer Materials, Center for Advanced Low-dimension Materials, College of Material Science and Engineering, Donghua University, Shanghai 201620, China; suchao@yeah.net (C.S.); sunjiaxing5@sina.com (J.S.); zxj19910621@163.com (X.Z.); shenduan5@126.com (D.S.)

**Keywords:** poly(2-ethyl-2-oxazoline), hydrogen bonding, layer-by-layer assembly

## Abstract

The hydrogen-bonded polymer complex thin film of poly(2-ethyl-2-oxazoline) (PEOX) and poly(acrylic acid) (PAA) was fabricated with layer-by-layer (LbL) assembly. The film shows exponential growth at early stage and transfers to linear growth after 10 assembling cycles, and the stable thickness increment per assembling cycle in the linear region could be higher than 100 nm. The film growth should be related with polymer chain diffusion during LbL assembly. The effects of assembling time, rinsing time, temperature, pH value, concentration and molecular weight on the thin film growth were investigated. Increasing the assembly time, the temperature and the concentration is favorable to produce the thick film. Prolonging rinsing time is good for preparing smooth film. The film can be constructed below pH 4.5 while the prepared film will not completely dissolve until pH value elevates to 7.0. Molecular weight has a subtle effect on the PEOX/PAA film growth. The PEOX-PAA pair that has a big molecular weight contrast shows fast film growth in the linear region.

## 1. Introduction

Poly(2-oxazoline)s have an amide group on the side chain, and show analogy to polypeptides. The synthesis of poly(2-oxazoline)s with living cationic ring-opening polymerization was reported in 1960s [1,2,3,4]. But for a long time, the poly(2-oxazoline)s have not been paid much attention. In recent years, poly(2-oxazoline)s have been widely exploited for biomedical applications due to their biocompatibility [5,6,7,8], thermal responsive behavior [9,10,11] and easy chemical modification [12,13,14,15]. The living cationic ring-opening polymerization makes 2-oxazolines able to be polymerized into a well-defined polymeric structure, in which the block segment length and the end-group functionality can be finely adjusted [16]. In addition, properties of poly(2-oxazoline)s can be tuned by varying the side chain of the 2-oxazoline monomer, and hence hydrophilic, hydrophobic or amphiphilic properties can be easily accessed [17]. Interestingly, some poly(2-oxazoline)s, such as poly(2-ethyl-oxazoline), poly(2-isopropyl-oxaoline) and poly(2-propyl-oxaoline), exhibit a lower critical solution temperature (LSCT) in water, which has the potential to develop into thermo-responsive materials [18]. 

Poly(2-oxazoline)s were reported to form polymer complex with poly(carboxylic acid) basing on hydrogen bonding [19]. Polymer complex is a kind of compound that two different polymers aggregate together by multiple and cooperative secondary interactions, such as electrostatic force and hydrogen bonding. Decher et al. demonstrated a delicate method, layer-by-layer (LbL) assembly, to prepare polyelectrolyte complex thin film on solid substrate, in which a positively charged and a negatively charged polymer are alternately deposited with a controllable way [20,21,22]. Subsequently, hydrogen-bonded polymer complex thin films were also prepared with LbL assembly [23,24]. Up to now, LbL assembly has been considered to be a simple but effective approach to produce polyelectrolyte complex and hydrogen-bonded polymer complex thin films which shows applications in various areas [25,26], such as separation membrane [27], optical filter [28], chemical and biological sensor [29,30], and drug delivery [31,32,33]. 

To prepare hydrogen-bonded polymer complex thin film with LbL assembly, a polymer pair, one hydrogen bond donor and the other hydrogen bond acceptor, is needed. Poly(carboxylic acid)s and polyphenols are two main kinds of hydrogen bonding donor polymer. The polymers that can form polymer complex with polyphenol and poly(carboxylic acid) include poly(vinylpyrrolidone) (PVPON) [34], poly(vinylpyridine) (PVP) [35], poly(ethylene oxide) (PEO) [36], poly(acrylamide) (PAM) [37], poly(vinyl alcohol) (PVA) [38], cellulose ether [39], polybenzoxazine [40], polyaniline [23,41,42,43] and poly(oxazoline)s [44,45]. Among them, PVPON, PVP and PEO are commonly used as hydrogen bond acceptor polymers for LbL assembly [46]. Poly(oxazoline) is seldom selected as acceptor polymer for hydrogen bonding LbL assembly. Recently, Hoogenboom et al. studied LbL assembly of poly(2-(*n*-propyl)-2-oxazline) (PPOX) and tannic acid (TA) [47,48]. PPOX shows LCST at 25 °C. Above and below LCST, hydrogen bonded PPOX/TA film shows different growth behavior [47]. Caruso and coworkers reported the poly(2-oxazoline)s for the assembly of low-fouling polymer capsules with redox-responsive behavior and intercellular degradability [49]. Sequential deposition of thiol-containing PEOX/poly(methacrylic acid) multilayers onto silica particle templates and crosslinking yielded stable capsules after removing the silica templates [50]. Sun et al. demonstrated that the film prepared with partially hydrolyzed PEOX and PAA shows healable and anti-fouling properties [51]. 

As a promising biomedical material, poly(2-oxazoline)s should be further explored on the platform of LbL assembly. In this work, LbL assembly of a poly(2-oxazoline), PEOX, and PAA was deeply studied. We investigate the effect of dipping time, temperature, pH value, concentration and molecular weight, on the PEOX/PAA film growth, compare the film with other hydrogen-bonded film, such as PEO/PAA and PVPON film, and discuss the film growth mechanism. 

## 2. Experimental Section

### 2.1. Materials

**Scheme 1:**
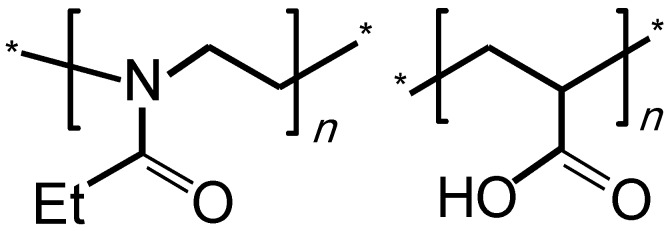
The chemical structures of poly(2-ethyl-2-oxazoline) (PEOX) and poly(acrylic acid) (PAA) molecules.

Poly(2-ethyl-2-oxazoline)s (PEOX, *M*_w_ = 50,000 and 500,000) were obtained from Alfa Aesar (Tianjin, China). Poly(acrylic acid) (PAA, *M*_w_ = 450,000) and PAA sodium salt solution (35%, *M*_w_ = 15,000) were purchased from Sigma-Aldrich (Shanghai, China). Both PEOX and PAA were used as received without further purification. Chemical structures are shown in Scheme 1.

### 2.2. Film Fabrication

The LbL assembly of PEOX and PAA was conducted on silicon or quartz substrate with an automatic machine (Kejing Auto-Instrument Co., Ltd., Shenyang, China). The surface of the substrate was cleaned by the following procedure: (a) immersing the substrate in boiling H_2_SO_4_/H_2_O_2_ (7/3, *V*/*V*) for 30 min (“piranha”: this solution is extremely corrosive); (b) washing with deionized (DI) water several times; (c) drying with a nitrogen flow. Generally, the substrates were alternately dipped into PEOX solution and PAA solution for 4 min with an interval of three rinses (1 min each). The pH values of the assembling solutions and rinsing solutions were adjusted to the same with HCl or NaOH solution under monitoring of a pH meter (Delta. 320, Mettler Toledo, Switzerland) After assembling, the wet film was blown dry with nitrogen flow, and then stored in the desiccator. The film is marked as (PEOX/PAA)_n_, where *n* indicates that the film was fabricated by *n* assembly cycles. 

### 2.3. Characterization

UV-visible spectra of the solutions and the thin films were recorded with UV-2550 spectrophotometer (Shimadzu, Japan). For the solution measurement, we applied standard quartz cuvette. For the thin film measurement, the films were deposited on the quartz substrates. The hydrogen-bonded thin films deposited on cleaned silicon wafers were directly measured on a FT-IR spectrometer (Nicolet 8700, ThermoFisher, Waltham, MA, USA) with a spectral resolution of 4 cm^−1^. PEOX powder and PAA powder were milled with KBr separately and pressed into standard discs for IR measurement. The thin film morphology characterization was conducted on an AFM (Agilent 5500, Agilent, Santa Clara, CA, USA) instrument under the tapping mode in air at room temperature. A commercial silicon probe (model TESP-100) with a resonant frequency of ≈300 kHz was used to scan the film.

## 3. Results and Discussion

**Figure 1:**
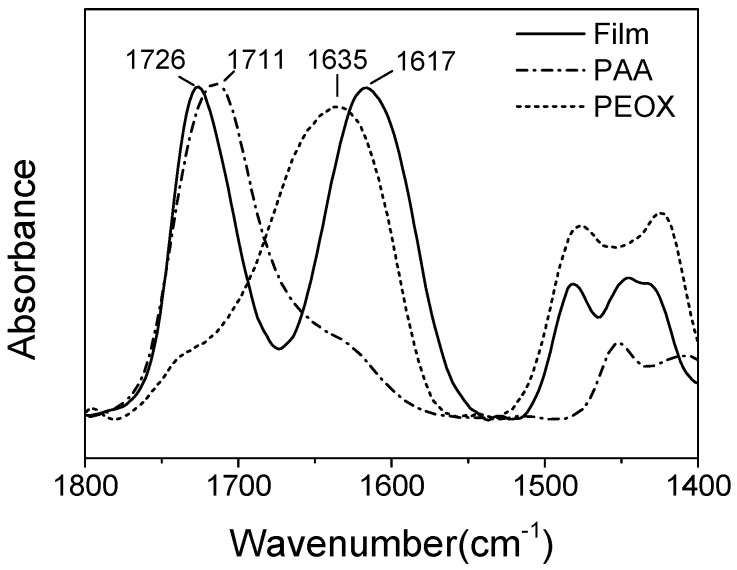
FT-IR spectra of PEOX, PAA and (PEOX/PAA)_20_ film (PEOX, *M*_w_ 50,000; PAA, *M*_w_ 450,000; pH 2.0; assembling time, 4 min; rinsing time, 1 min; and room temperature).

PEOX and PAA can form polymer complex based on hydrogen bonding [19,45]. PEOX and PAA were LbL assembled on silicon substrate, and the FT-IR spectrum of (PEOX/PAA)_20_ film was shown in Figure 1. For comparison, the spectra of pure PEOX and PAA powder were presented. 

PEOX/PAA film shows the C=O (carboxylic acid) stretching peak of PAA at 1726 cm^−1^ while the C=O stretching peak (amide) of PEOX was at 1617 cm^−1^. The pure PAA exhibits the C=O stretching peak at 1711 cm^−1^. The pure PEOX presents the C=O stretching peak at 1635 cm^−1^. The C=O stretching of free COOH generally is located at 1740–1745 cm^−1^ [52]. In pure PAA, there are many different hydrogen bonding modes among COOH groups, so the C=O stretching band becomes broad and asymmetric, and locates at low frequency compared with that of free COOH. When PAA was LbL assembled with PEOX, the hydrogen bonding would be mainly between PEOX and PAA instead of the hydrogen bonding among COOH groups of PAA. One phenomenon, C=O stretching vibration peak of PAA (1726 cm^−1^) in the PEOX/PAA film shows blue shift compared with that in pure PAA powder (1711 cm^−1^), but shows red shift compared with that in free COOH (1740–1745 cm^−1^). In pure PEOX powder, there is no hydrogen bonding. The C=O stretching vibration peak of PEOX (1617 cm^−1^) in PEOX/PAA film showed red-shift compared with pure PEOX powder (1635 cm^−1^). IR spectra demonstrated that PEOX and PAA were LbL assembled to fabricate the thin film based on hydrogen bonding.

### 3.1. Film Growth Mode

**Figure 2:**
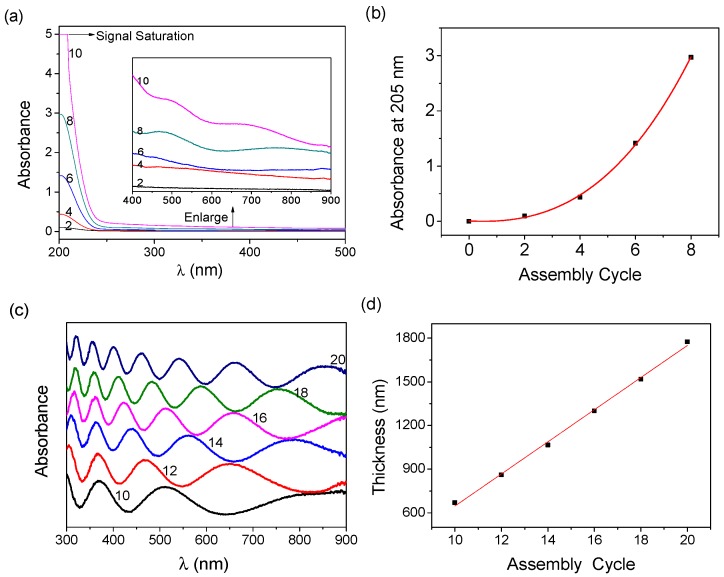
UV-visible spectroscopy was applied to monitor the PEOX/PAA film growth: (**a**) UV-vis spectra of the film as the number of assembling cycle increased from 2 to 10, spectroscopic range 200–500 nm; (**b**) The absorbance of the film at 205 nm as a function of assembly cycle; (**c**) UV-vis spectra of the film as the number of assembling cycle increased from 10 to 20, spectroscopic range 300–900 nm, for clearance spectra were intentionally overlaid; (**d**) The film thickness as a function of assembly cycle. (PEOX, *M*_w_ 50,000, 1.0 mg/mL; PAA, *M*_w_ 450,000, 1.0 mg/mL; pH 2.0; assembling time, 4 min; rinsing time, 1 min; and room temperature).

UV-visible spectroscopy was applied to monitor PEOX/PAA film growth as a function of assembly cycle (i.e., the number of bilayer), as shown in Figure 2. As the number of assembling cycles increased, the absorbance of the film would quickly increase (Figure 2a). According to the Lambert-Beer law, the chromophore absorbance of the film is proportional to the film thickness [53]. The quick absorbance increasing reflects that the fast film growth. The absorbance of the film at 205 nm as a function of assembly cycle was shown in Figure 2b. The film growth was non-linear, exponential-like. However, as the number of the assembling cycles increased to 8, the absorbance of the film would be about 3.0, close to saturation. For the film prepared with 10 assembling cycle, the absorbance peak became flatten, and the absorbance signal was saturated (Figure 2a). After 8 assembly cycle, the absorbance signal of the film would be saturated and we could not monitor film growth any more by using the absorbance information. The door closed but a window opened. As shown in the insetting plot of Figure 2a, when the absorbance peak became saturated, the thin film would show oscillations on the spectrum. These oscillations are named as Fabry-Pérot fringes, caused by light interference of the thin film [54,55]. When the thin film is smooth, its thickness gets to the level of visible light, and there is an obvious refractive index contrast between the film and the substrate, the film will cause the light interference and hence there are oscillations on the spectra. As shown in Figure 2c, after 8 assembling cycles the films showed Fabry-Pérot fringes on the spectra. 

Fabry-Pérot fringes on UV-visible spectra can be used to estimate the film thickness, according to the equation as below [54,55]:

(1) $$d=\frac{m\lambda_{k}\lambda_{k+m}}{2n\left(\lambda_{k}-\lambda_{k+m}\right)}$$

where d is the film thickness; n is the effective refractive index of the film (n = 1.5); $\lambda_{k}$ and $\lambda_{k+m}$ are the peak positions of the kth and (k+m)th interference on the spectrum; and m is the difference of the level between these two peaks. According to Equation (1), the thickness of the film can be calculated. The film thickness as a function of assembling cycle (from 10 to 20) was shown in Figure 2d. When the number of the assembling cycle exceeded 10, the film thickness would show linear growth. By combination of the experimental results from Figure 2b,d, the growth behavior of (PEOX/PAA)_*n*_ film can be described. Before 10 assembling cycles, the film exhibits exponential growth while after 10 assembling cycles, the exponential growth transfers to linear growth.

The film growth mechanism is a key fundamental issue of LbL assembly. At the early stage of LbL assembly study, the linear growth was mainly reported and the thickness increment per assembling cycle was less than 10 nm [56]. However, as the researching going on, the systems that contain weak and highly hydrated polyelectrolyte exhibit exponential growth, such as poly(l-lysine)/alginate, poly(l-lysine)/hyaluronic, poly(l-lysine)/poly(l-glutamic acid), and poly(allylamine hydrochloride)/poly(l-glutamic acid) [57,58,59]. Picart et al. put forward a “in and out diffusion” mechanism to explain the exponential growth, and provided direct molecular evidence [60]. The further investigation found that after a certain number of assembling cycle, the exponential growth would transfer to linear growth [61]. Decher considered that LbL was a fuzzy assembly [62]. Though the materials are LbL deposited, the laminated structure is not clear, and the interpenetration happens due to diffusion inside the film. Interpenetration degree depends on the assembling pair and processing conditions. Also there is an opinion that non-linear growth is related with surface roughness [63,64]. Haynie et al. put forward island model and dendritic model to explain the non-linear growth of LbL film [64].

For hydrogen-bonded PEOX/PAA film, after 10 assembling cycles the film thickness transfers from exponential growth to linear growth. The stable thickness increment per assembling cycle is more than 100 nm, which is higher than the sum of gyration radii [65] of PEOX and PAA (Supplementary Materials). The simple surface adsorption and functional group compensation cannot be used to explain the growth of the hydrogen-bonded PEOX/PAA film. Schlenoff commented that for LbL assembled polyelectrolyte film the amounts of polyelectrolyte were not adsorbed in their thermodynamic limit; and linear or exponential growth represented different ends of a continuous spectrum of diffusion [66]. 

Our previous work showed that hydrogen-bonded PVPON/PAA film have the same kind of growth behavior, before 10 assembly cycles exponential growth while after 10 assembly cycle liner growth [54]. We designed experiments to prove that polymer chains are diffusing in and out during the construction of hydrogen-bonded PVPON/PAA complex thin film with LbL assembly (Supplementary Materials Figure S1). PAA was labeled with a fluorescence dye, fluorescein amine (FAM), which was marked as PAA-FAM. The hydrogen-bonded (PVPON/PAA)_20_ was immersed into PAA-FAM solution, and we observed that the absorbance of PAA-FAM and film thickness both increase. The outer layer of (PVPON/PAA)_20_ film is PAA. This indicates that the PAA chain diffuses into the film, and otherwise the film thickness will not increase. Further (PVPON/PAA)_20_ film was immersed into PAA-FAM solution for 48 h, and then in PVPON solution. After immersion in PVPON solution, we observed that the absorbance of PAA-FAM decreased but film thickness increased. This demonstrated that PVPON diffuse in while PAA diffuse out. Hydrogen bonding dominated LbL assembly system is a dynamic process, during which there are polymers that moved from the solution into the film and also there are polymers that moved from the film into the solution. The film thickness increment is due to the mass of polymers that moved into the film is more than that of the polymers that moved out from the film. We assume that the film growth mechanism of PEOX/PAA is similar to that of PVPON/PAA film. 

There are many controlling parameters for LbL assembly, such as molecular weight of assembling polymers, molecular weight contrast of polymer pair, assembling time, rinsing time, temperature, concentration, and pH value. We investigated the effects of these parameters on the film growth. How these factors affect the film thickness will help us deeply understand the mechanism of the growth of PEOX/PAA film.

### 3.2. Assembling Time and Rinsing Time

**Figure 3:**
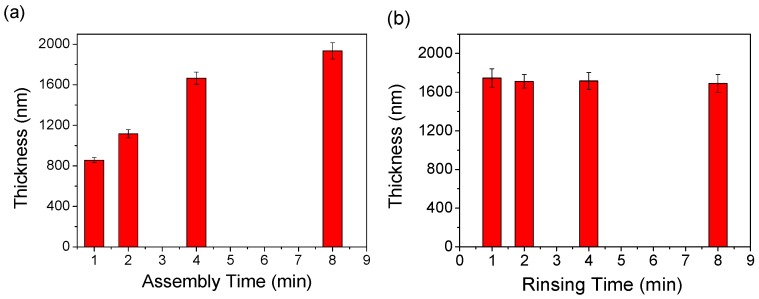
Thickness of (PEOX/PAA)_20_ film: (**a**) the film prepared with different assembling time, rinsing time fixed to be 1 min; (**b**) different rinsing time, assembling time fixed to be 4 min. (PEOX, *M*_w_ 50,000, 1 mg/mL; PAA, *M*_w_ 450,000, 1 mg/mL; pH 2.0; and room temperature).

We changed the assembling time and fixed the rinsing time (1 min each, three times). The thickness of (PEOX/PAA)_20_ prepared with different assembling time was shown in Figure 3a. As the assembling time was prolonged, the film became thick. 

The assembling time was fixed for 4 min while the rinsing time varied, and the thickness of the film prepared with different rinsing time was shown in Figure 3b. The film thickness did not show dependence on rinsing time, but rinsing time affects the surface morphologies of the thin film (Supplementary Materials, Figure S2). With the shorter rinsing time, there are many complex bumps on the surface. As the rinsing time becomes long, the bumps will disappear and the film will become smooth. 

The film thickness shows strong dependence on assembly time, which supports the proposition that the film growth is related with polymer chain diffusion. During LbL assembly of PEOX and PAA, polymer chains are adsorbed on the surface and then the adsorbed polymer chains diffuse inwardly. The diffusion inside the film is much slower than surface adsorption. The diffusion time scale in the assembled film determines the dependence of the film growth on the assembly time. 

Rinsing during LbL assembly removes loosely attached polymer chains and is beneficial for improving the homogeneity and the flatness of the film. Our previous work investigated the effect of rinsing time on LbL assembly of PVPON and PAA [67]. As the rinsing time became long, the PVPON/PAA film became smooth. On the one hand, rinsing gets rid of the loosely bounded polymers, and on the other hand, the longer rinsing time is helpful for chain rearrangement and hydrogen bond pairing, which increases the smoothness of the assembled film. 

### 3.3. Temperature

**Figure 4:**
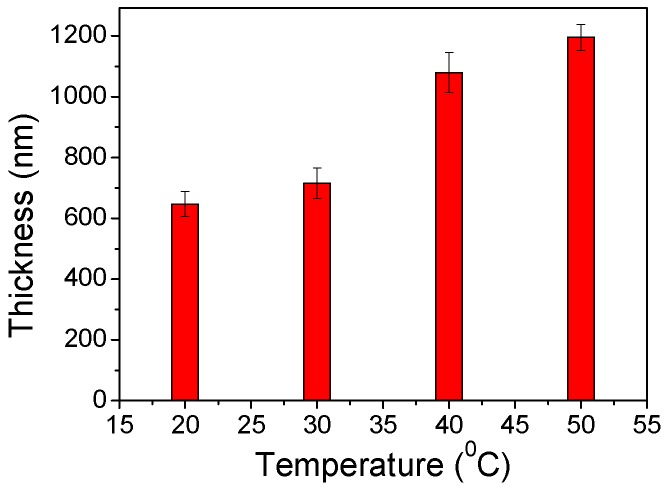
The thickness of (PEOX/PAA)_10_ film prepared with different temperature. (PEOX, *M*_w_ 50,000, 1 mg/mL; PAA, *M*_w_ 450,000, 1 mg/mL; pH 2.0; assembling time, 4 min; and rinsing time, 1 min).

PEOX is a thermo-responsive polymer and shows low-critical solution temperature (LCST). The LCST of PEOX was reported to be 65 °C [68]. To avoid phase separation of polymer chain in the solution, the LbL assembly of PEOX and PAA is performed far below the LCST point of PEOX, where PEOX chain and PAA chain are both in well-dissolved state. The UV-Visible spectrometer was utilized to characterize the film prepared at different temperature. As temperature increased, there would be more Fabry-Pérot fringes on UV-Visible spectra (Supplementary Materials, Figure S3). The thicknesses of (PEOX/PAA)_10_ films fabricated at 20, 30, 40, and 50 °C were shown in Figure 4. As temperature elevated, the film would become thick. 

Caruso et al. reported that temperature greatly affect the growth of the hydrogen-bonded PNIPAAM/PAA film. The film fabricated at 30 °C was thicker than the films prepared at 21 or 10 °C [69]. Our previous work investigated the temperature dependence on the thickness of PVPON/PAA film [70]. PVPON/PAA film thickness showed strong dependence on temperature. Like the PVPON/PAA system, PEOX/PAA exhibited strong dependence on temperature too. Straightforwardly, temperature elevation makes diffusion accelerate, and hence in the fixed time period, more polymers will be transferred from the assembling solution to/into the assembled film and the film growth will become fast.

### 3.4. pH Value

PAA is a weak polyelectrolyte. As pH value elevates, the ionization degree of PAA will increase, which affects its complexation behavior with hydrogen bonding acceptor polymer [71]. At low pH value, the mixed solution of PAA and PEOX is cloudy and the precipitation is produced. As pH value gradually increased from 1.0 to 4.5, the mixed solution would change from cloudy to transparent (Supplementary Materials, Figure S4). This indicated that when pH value was higher than pH 4.5, PEOX and PAA could not form a solid-like polymer complex. 

The LbL assembly of PEOX and PAA was conducted at different pH values. Fabry-Pérot fringes and chromophore absorbance on UV-Visible were both applied to monitor the film thickness. As pH values increased, the number of Fabry-Pérot fringes would decrease (Supplementary Materials, Figure S5), indicating that the film became thin. When pH value was elevated to 3.5, (PEOX/PAA)_20_ film became very thin and there were no Fabry-Pérot fringes present. The chromophore absorbance was utilized to monitor the film growth (Supplementary Materials Figure S6). At pH 3.5, 3.7 and 4.0, we could observe the slow film growth. When the pH value was elevated to 4.5, the film growth was extremely slow. We considered that pH 4.5 was the onset pH value to forbid the LbL assembly of PEOX and PAA. 

**Figure 5:**
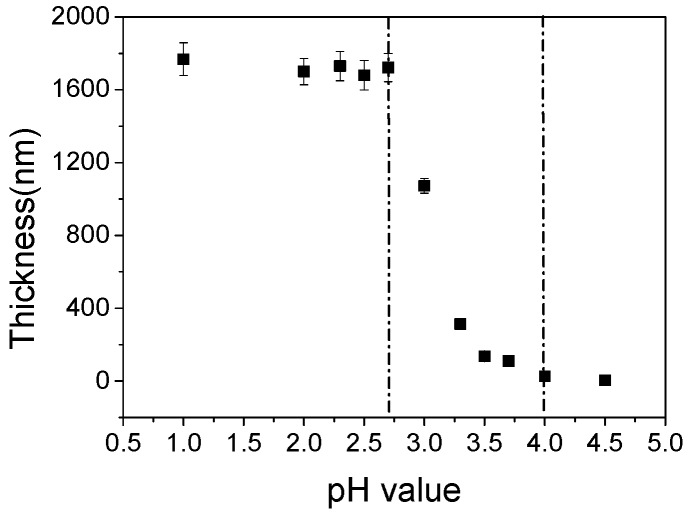
Thickness of (PEOX/PAA)_20_ as a function of pH value. (PEOX, *M*_w_ 50,000, 1 mg/mL; PAA, *M*_w_ 450,000, 1 mg/mL; assembling time, 4 min; rinsing time, 1 min; and room temperature).

The film thickness as a function of pH value was shown in Figure 5. In the pH region from 1.0 to 2.7, relatively thick film could be prepared and the film thickness showed small variation as pH values changed. However, when the pH value was higher than 2.7, the film thickness would decrease rapidly as the pH value elevated. When the pH value was higher than 4.5, the film growth would be almost restricted, i.e., PEOX and PAA could not be LbL assembled to prepare thin film. The effect of pH on other hydrogen-bonded LbL assembly systems, such as PVPON/PAA and PEO/PAA, has been studied [72,73]. PEOX/PAA film showed the same pH dependence behavior like PVPON/PAA and PEO/PAA films. As the pH values elevated, PVPON/PAA and PEO/PAA cannot be produced with LbL assembly. The critical pH values to forbidding the growth of PVPON/PAA and PEO/PAA films were reported to be 4.0 and 3.5, respectively [72,73].

**Figure 6:**
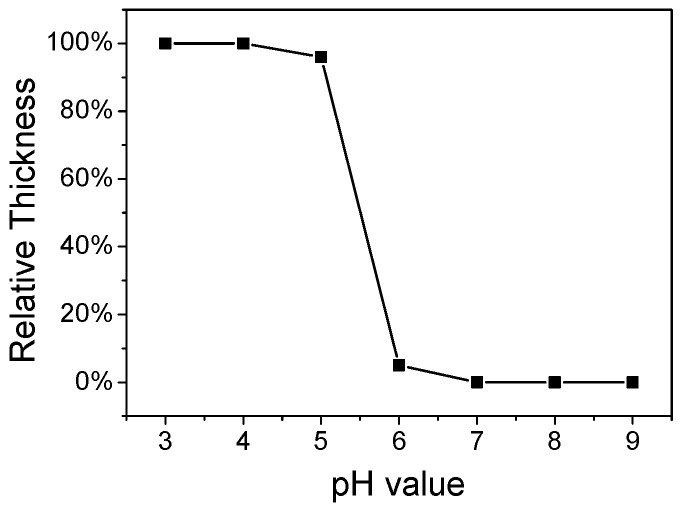
Decomposition of the film in different pH value solution in 6 h. (The original films are fabricated: PEOX, *M*_w_ 50,000, 1 mg/mL; PAA, *M*_w_ 450,000, 1 mg/mL; pH 2.0; assembling time, 4 min; rinsing time, 1 min; and room temperature).

Like other hydrogen-bonded films, PEOX/PAA thin films will be decomposed in the higher pH value solution. In the high pH value environment, the ionization of PAA will make the hydrogen bond break, which results in the decomposition of PEOX/PAA film. To determine the decomposition pH value, the prepared (PEOX/PAA)_20_ films were immersed in different pH value solution for 6 h. The thickness variation as a function of pH value was shown in Figure 6. When pH value was lower than 5.0, the film did not show a decrease in thickness. After being immersed into the solution of pH 5.0 for 6 h, the film thickness only exhibited a slight thinning. When the pH value was elevated to 6.0, the thickness showed a great decline. At pH 7.0, we observed the complete disintegration of the hydrogen-bonded PEOX/PAA film in 6 h. 

When the pH value was elevated to 4.5, LbL assembly of PEOX and PAA would be forbidden. However, the prepared PEOX/PAA film did not dissolve at pH 4.5. For the complete dissolution of PEOX/PAA film, the pH value needed to be higher than 7.0. So there was a pH discrepancy between the critical pH value for the film formation and that for the film disintegration. For PEOX/PAA bulk complex, the pH value discrepancy between formation and dissolution was reported [74]. The hydrogen-bonded PVPON/PAA film, also exhibited the pH discrepancy of formation and disintegration [72]. The pH discrepancy for hydrogen bonded film formation and disintegration is considered to be related to kinetic reasons [74]. 

### 3.5. Concentration

Concentration gradient is the driven force for diffusion. The growth of PEOX/PAA film is related to polymer chain diffusion. So the concentration of the assembling solution should affect the thickness of PEOX/PAA film [75]. In the above sections, the concentration was calculated in mg/mL. PEOX concentration and PAA concentration were both fixed at 1.0 mg/mL. In this section, the concentration is calculated with the molar concentration (mol/L or mmol/mL) of the repeat unit of the polymer. Two sets of experiments have been designed to investigate the concentration effect on the film thickness: (1) keeping the concentration ratio unchanged, but PEOX and PAA concentration both increase; (2) fixing the total concentration, but the concentration ratio between PEOX and PAA varies. 

**Figure 7:**
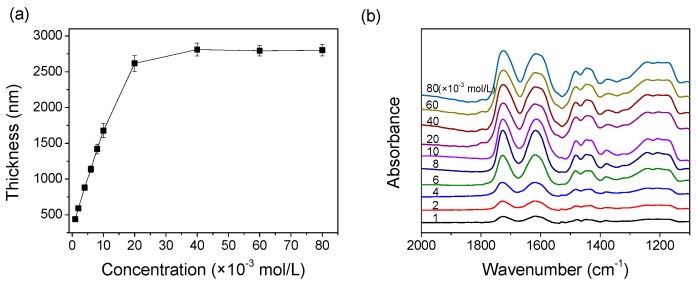
(PEOX/PAA)_20_ prepared with different concentration: (**a**) thickness of the film as a function of the concentration; (**b**) FT-IR spectra of the films, for clearance the spectra are intentionally overlaid. ([EOX]/[AA] = 1; PEOX, *M*_w_ 500,000; PAA, *M*_w_ 450,000; pH 2.0; assembling time, 4 min; rinsing time, 1 min; and room temperature).

In the first set of experiments, the concentration ratio between PEOX and PAA was fixed at 1.0, and PEOX concentration and PAA concentration both gradually increased from 1.0 × 10^−3^ to 8.0 × 10^−2^ mol/L. The film thickness as a function of the concentration was shown in Figure 7a. As the concentration increased, the PEOX/PAA film became thick. However, when the concentration was elevated to 3.0 × 10^−2^ mol/L (PEOX, 2.97 mg/mL; PAA, 2.16 mg/mL), the thickness increasing tendency stopped and as the concentration further elevated the film thickness remained stable. 

The FT-IR was also applied to characterize the PEOX/PAA films prepared with different concentration, as shown in Figure 7b. As the concentration of assembling solution increased, the vibration absorbance band would become strong, indicating the film grew thick. As demonstrated by Figure 1, the absorbance bands at 1617 and 1726 cm^−1^ are assigned to C=O stretching vibrations of PEOX and PAA, respectively. So the absorbance ratio between 1617 and 1726 cm^−1^ (A_1617_/A_1726_) can reflect the composition ratio between PEOX and PAA in the film. As the concentration increased, A_1617_/A_1726_ would show very small variation (Supplementary Materials, Figure S7). This indicated that when fixing the concentration ratio of PEOX and PAA assembling solutions, PEOX and PAA composition ratio inside the LbL film would keep stable.

For the second set of the concentration experiments, the total concentration of PEOX and PAA was fixed at 1.0 × 10^−2^ mol/L, but the concentration ratio changed. PEOX solution and PAA solution with different concentration ratio ([EOX]/[AA] = 1/9, 2/8, 3/7, 4/6, 5/5, 6/4, 7/3, 8/2, and 9/1) were mixed (Supplementary Materials Figure S8). When the concentrations of PEOX and PAA solution were close, the mixed solution would be very cloudy. PEOX and PAA would be easy to form a polymer complex when their molar concentrations were close. 

**Figure 8:**
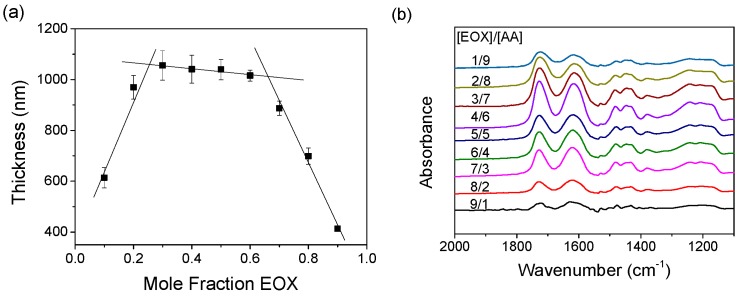
(PEOX/PAA)_20_ prepared with different concentration ratio: (**a**) thickness of the film as a function of the concentration ratio (log([EOX]/[AA])); (**b**) FT-IR spectra of the films. ([EOX] + [AA] = 1.0 × 10^−2^ mol/L; PEOX, *M*_w_ 500,000; PAA, *M*_w_ 450,000; pH 2.0; assembling time, 4 min; rinsing time, 1 min; and room temperature).

The LbL assembled films were prepared with different concentration ratio ([EOX]/[AA] = 1/9, 2/8, 3/7, 4/6, 5/5, 6/4, 7/3, 8/2, and 9/1). The film thickness showed dependence on the concentration ratio of PEOX and PAA. To manifest this effect, a plot was drawn with log([EOX]/[AA]) as the abscissa while with the film thickness as the ordinate, as shown in Figure 8a. Figure 8a clearly demonstrated that when the concentration of PEOX was close to that of PAA, the film growth would be fast. When the ratio was far away from the stoichiometric point of hydrogen bonding, the film growth would become slow. 

Besides, the films prepared with different concentration ratio were characterized by using FT-IR spectrometer in Figure 8b. As above mentioned, the ratio between the absorbance at 1617 cm^−1^ (A_1617_) and the absorbance at 1726 cm^−1^ (A_1726_) can reveal the composition of PEOX and PAA in the film. As the concentration ([EOX]/[AA]) ratio of assembling solution increased, A_1617_/A_1726_ of the film would become high, indicating that the composition of PEOX in the film increased (Supplementary Materials, Figure S9). Therefore, we can adjust the film composition by changing the concentration ratio of the assembling solution. 

### 3.6. Molecular Weight

The molecular weight affects the growth of hydrogen-bonded film [76]. PEOX and PAA with different molecular weight were used for LbL assembly to prepare thin films. Molecular weight has a subtle effect on PEOX/PAA film thickness. When assembly cycle number was low, the high molecular weight pair produces thick film (Supplementary Materials, Figure S10). 

As above mentioned, before the 10th assembly cycle, PEOX/PAA film showed exponential growth mode and after the 10th assembly cycle, the film transferred to linear growth. From 10 to 20 assembly cycles, the films prepared with different molecular weight all showed linear growth. The thickness increment per assembly cycle in linear region reflects the diffusion equilibrium under a certain condition. So we pay more attention to thickness increment per assembly cycle in the linear region. The film growth per assembly cycles of the films exhibits the sequence: PEOX_500k_/PAA_15k_ > PEOX_50k_/PAA_450k_ > PEOX_50k_/PAA_15k_ > PEO_500k_/PAA_450k_. It meant that molecular weight affects the film growth, but the more important was molecular weight contrast between two polymers. For the film PEOX_500k_/PAA_450k_, the molecular weight of PEOX and PAA are very close, and its growth is slowest among them in the linear region. However, the film PEOX_500k_/PAA_15k_ has big molecular weight contrast and shows the fastest growth among them in the linear growth region. 

**Figure 9:**
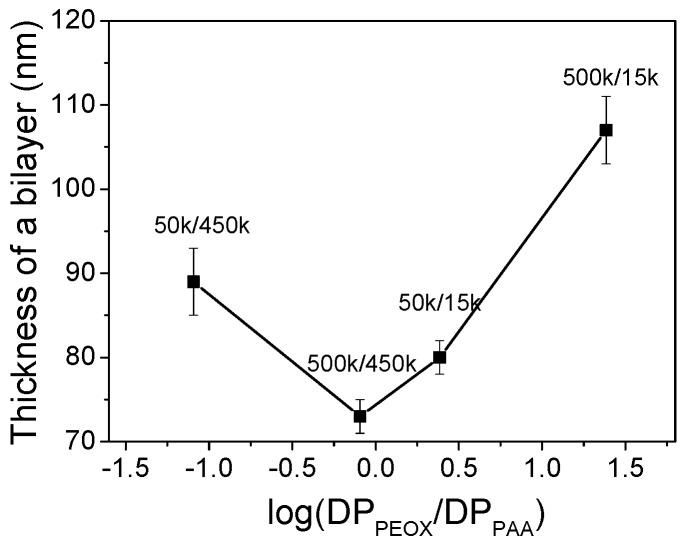
Thickness increment of PEOX/PAA film per assembly cycle in the linear growth region as a function of molecular weight contrast. The molecular weight contrast is expressed as log(DP_PEOX_/DP_PAA_), where DP_PEOX_ and DP_PAA_ are polymerization degree of PEOX and PAA respectively. (PEOX, [EOX] = 1.0 × 10^−2^ mol/L; PAA, [AA] = 1.0 × 10^−2^ mol/L; pH 2.0; assembling time, 4 min; rinsing time, 1 min; and room temperature).

To manifest the effect of the molecular weight contrast, i.e., the polymer chain length contrast, we plotted the film thickness in the linear region as a function of log(DP_PEOX_/DP_PAA_) (DP is degree of polymerization), as shown in Figure 9. When two polymers’ molecular weight was close, the film growth would be relatively slow. However, when the molecular weight contrast became large, either PEOX was much bigger than PAA or PAA was much bigger than PEOX, and the growth would be relatively fast in the linear growth region. 

The diffusion of LbL assembly can be divided into two phases, one is the diffusion in the solution, and the other is the diffusion in the thin film. The diffusion in the solution is related with temperature, concentration, viscosity and polymer chain size. Under the condition of constant temperature, concentration and viscosity, the diffusion in the solution is affected by polymer chain size. However, the diffusion in the film is more complicated than that in the solution. This is because the thin film can be considered as gel state and the viscosity of the film is much higher than that of the solution. So the diffusion in the film is much slower than that in the solution. For the growth of an LbL film, the polymer diffusion in the thin film should be the critical influence factor. If the sizes of hydrogen bonding donor polymer and hydrogen bonding acceptor polymer match well, they will restrict each other and hence the diffusion in the film will become slow. However, if PEOX and PAA show big difference on size, the long chain can interact with several short chains, and cooperative hydrogen bonding will be weak and the diffusion in the film will become fast. Chain size matching degree affects cooperative hydrogen bonding and hence affects polymer chain diffusion in the film. 

## 4. Conclusions

PEOX and PAA can be LbL assembled to prepare thin films based on hydrogen bonding. PEOX/PAA film shows exponential growth before the 10th assembly cycle and after that the film transfers to linear growth. The film growth should be related with polymer chain diffusion in the assembled film and the assembling solution. The stable thickness increment per assembling cycle can exceed 100 nm in the linear region. Increasing the temperature, the assembling time and the concentration of the assembling solution will accelerate the film growth. Increasing the rinsing time is helpful to produce the smooth film. Changing the concentration ratio of PEOX solution and PAA solution can adjust composition of the resultant film. pH value affects the hydrogen bonding between PEOX and PAA. LbL assembly of PEOX and PAA can be LbL assembled to prepare thin film below pH 4.5 while complete dissolution needs a pH value higher than 7.0. 

Molecular weight has a subtle effect on PEOX/PAA film thickness. When assembly cycle number is low, the high molecular weight pair produces thick film. However, in the linear growth region, the pair that has the obvious molecular weight contrast shows the fastest growth. The stable thickness increment per assembly cycle shows strong dependence on molecular weight contrast. 

## Supplementary Materials

The following are available online at www.mdpi.com/2073-4360/9/8/363/s1.
