# Supplementary material for: Hydrogen-Bonded Polymer Complex Thin Film of Poly(2-oxazoline) and Poly(acrylic acid)
Source: Polymers (Basel). 2017 Aug 15;9(8):363. doi: 10.3390/polym9080363 (PMC6418716; doi:10.3390/polym9080363)
Supplement: Supplementary file 1 [file polymers-09-00363-s001.pdf]

Supplementary Materials: Hydrogen-Bonded Polymer Complex Thin Film of Poly(2-oxazoline) and Poly(acrylic acid)

Chao Su, Jiaxing Sun, Xuejian Zhang, Duan Shen and Shuguang Yang

The estimation of R_g

Chain dimensions are often expressed in terms of the characteristic ratio, k, which is defined by:

$$h^2 = k\frac{M}{m_0}l^2$$

since $h^2 = 6{R_g}^2$,

$${R_g}^2 = k\frac{M}{6m_0}l^2$$

where h^2 is the mean-square unperturbed end-to-end distance, ${R_g}^2$ is the mean-square unperturbed radius of gyration. M is the molecular weight, m_0 is the average molecular weight per backbone bond of length l.

For PEOX

When M = 50k, $R_{g(PEOX)} = 54.6$ Å ;

When M = 500k, $R_{g(PEOX)} = 172.7$ Å

For PAA

When M = 15k, $R_{g(PAA)} = 36.1$ Å ;

When M = 450k, $R_{g(PAA)} = 299.3$ Å

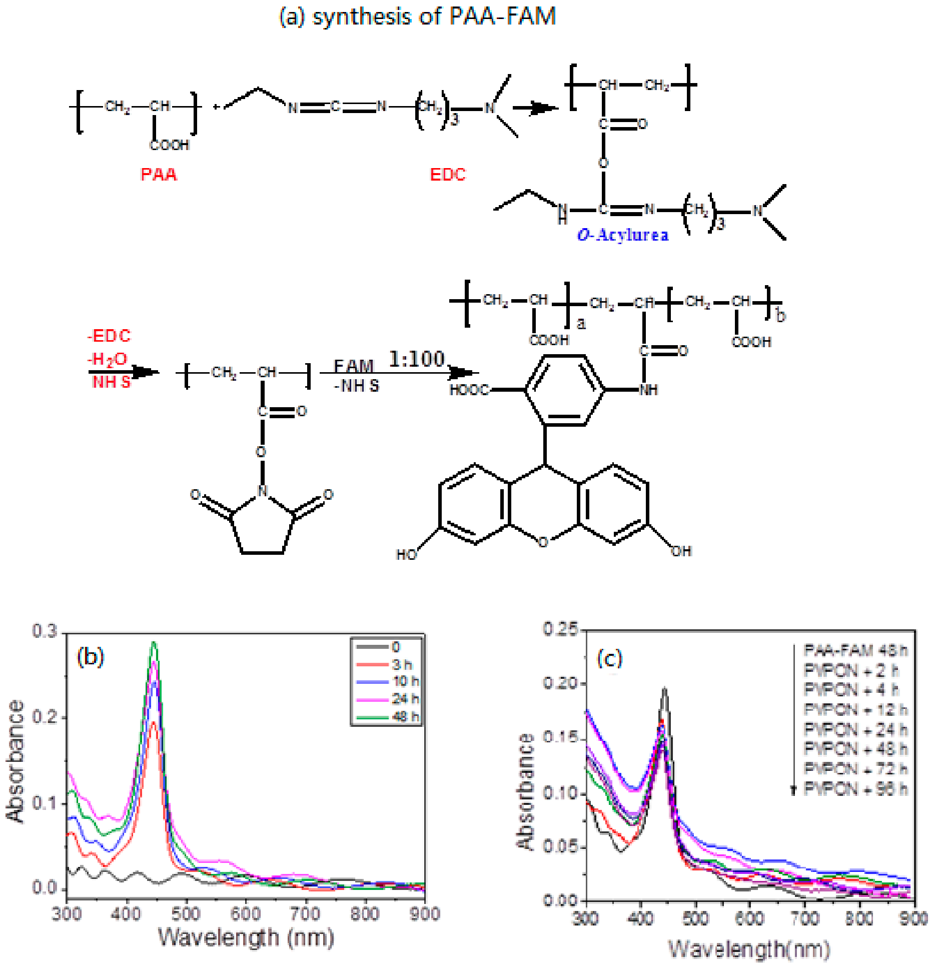

Figure S1. (**a**) The scheme of PAA-FAM synthesis; (**b**) UV-Visible spectra: one $(PVPON/PAA)_{20}$ film immersed into PAA-FAM solution for different time; (**c**) UV-Visible spectra: the other $(PVPON/PAA)_{20}$ film immersed into PAA-FAM solution for 48 h, and then immersed into PVPON solution for different time.

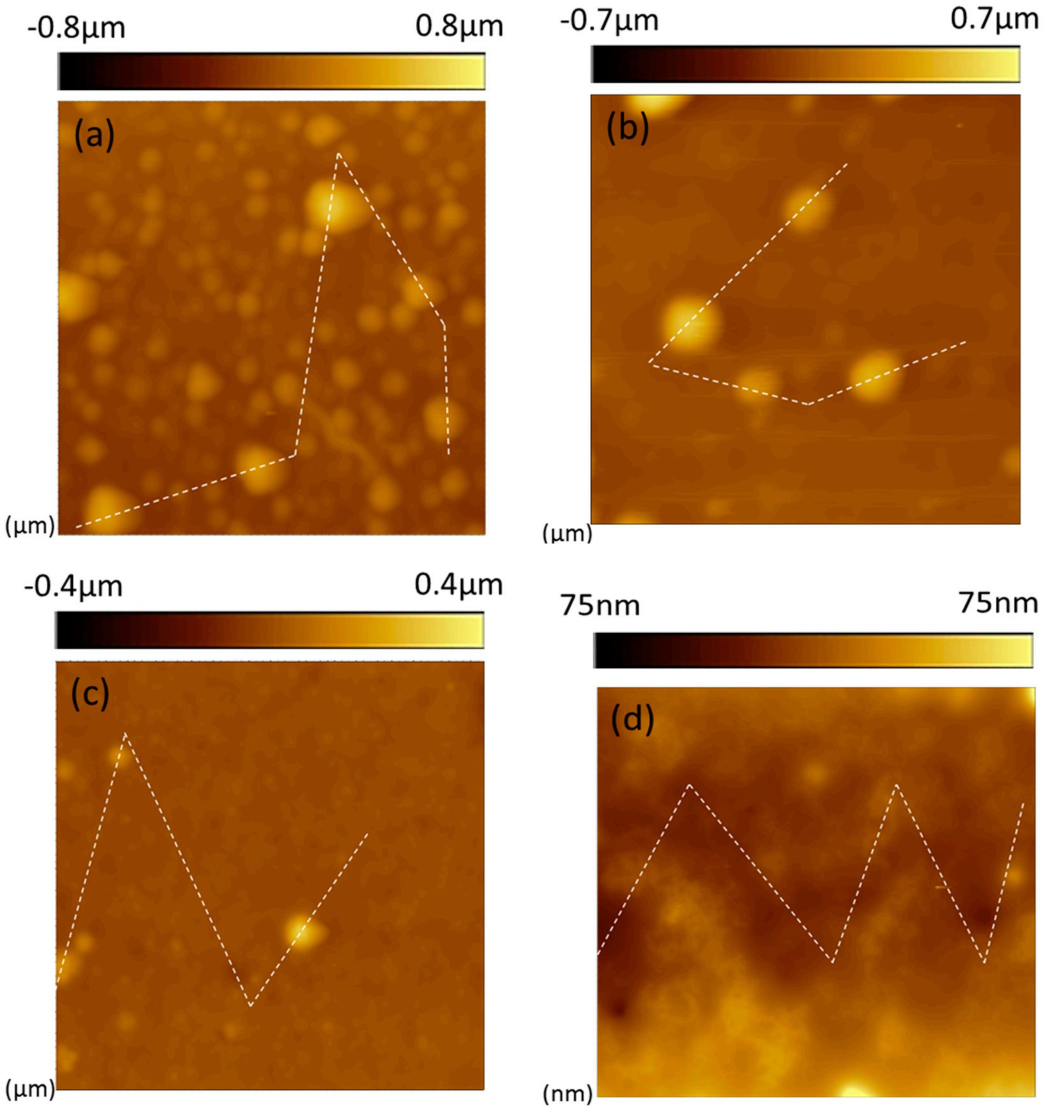

Figure S2. AFM images (30 μm × 30 μm) and the surface roughness of the (PEOX/PAA)20 film fabricated with different rinsing time. From (**a**) to (**d**): rinsing time is 1, 4, 8, and 16 min respectively.

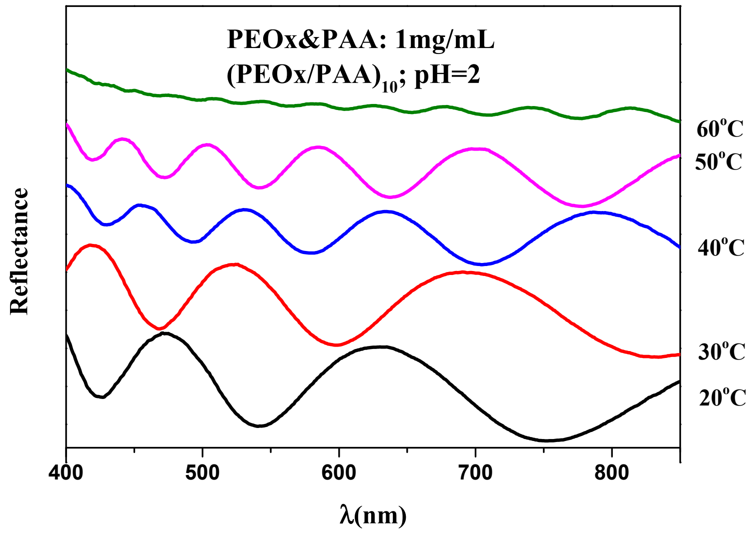

Figure S3. UV-visible spectra of the film fabricated at different temperature.

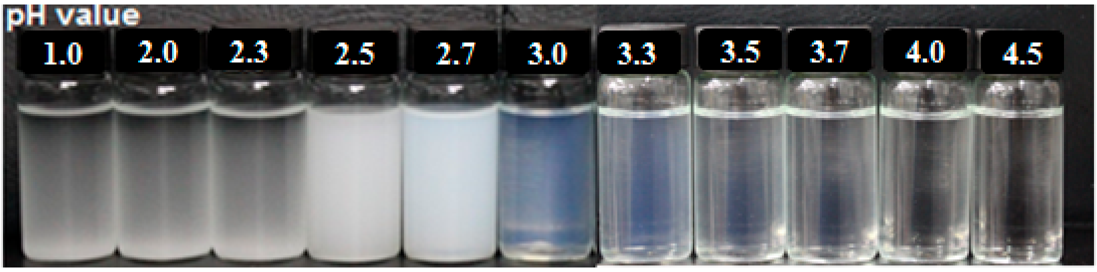

Figure S4. The photographs of the mixed solution of PEOX and PAA at different pH values.

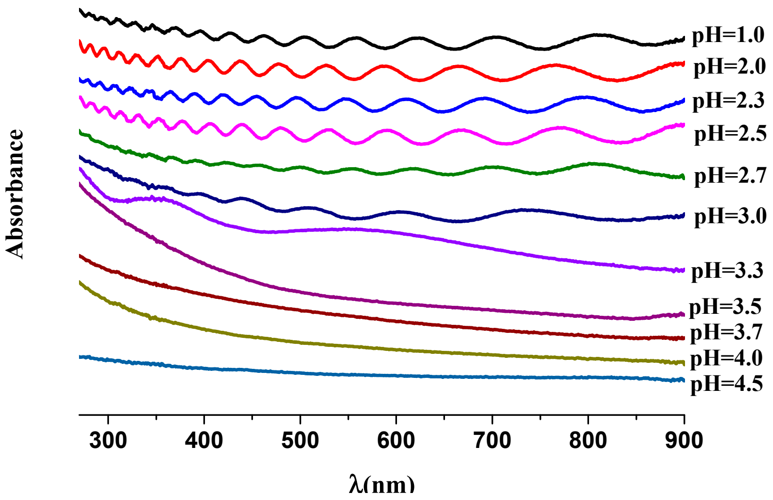

Figure S5. UV-visible spectra of $(PEOX/PAA)_{20}$ film prepared with different pH.

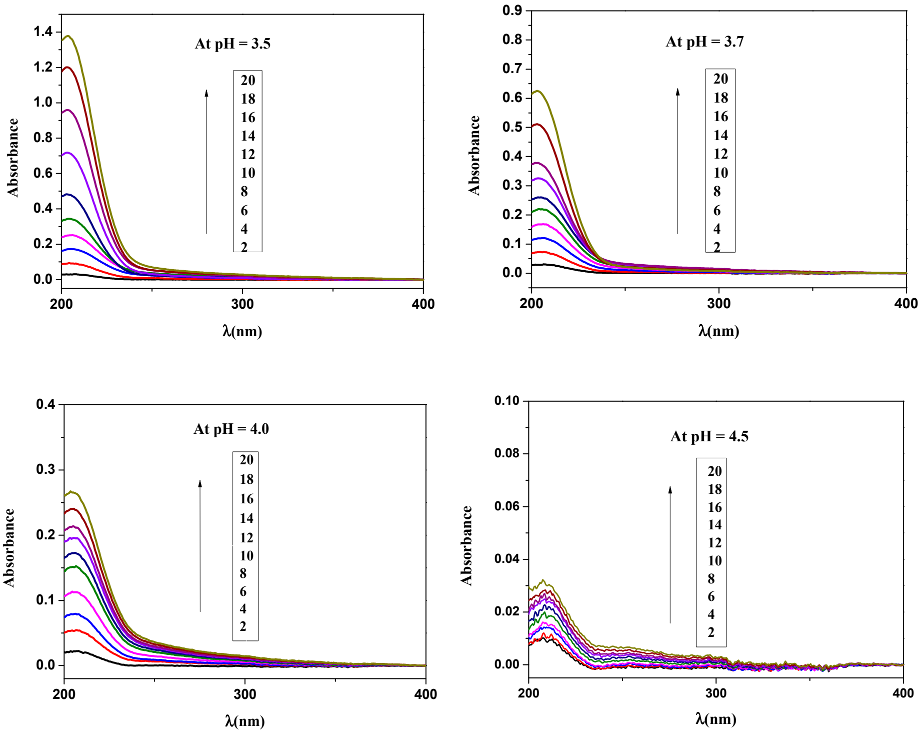

Figure S6. UV-visible spectroscopy monitoring film growth at different pH values: (**a**) pH 3.5; (**b**) pH 3.7; (**c**) pH 4.0; (**d**) pH 4.5.

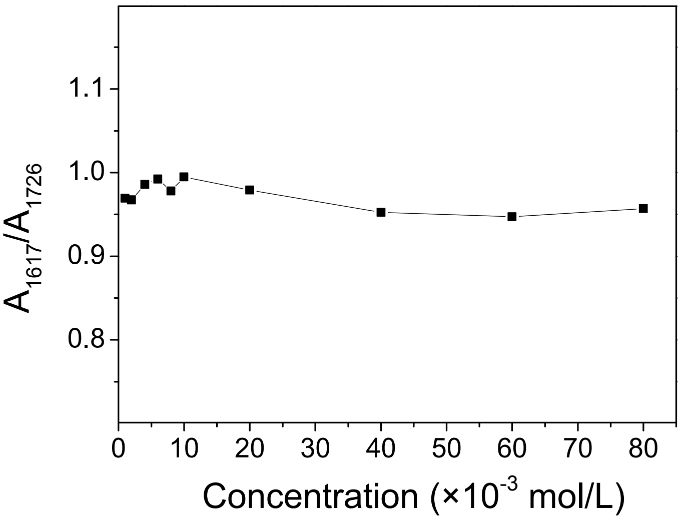

Figure S7. A_{1617}/A_{1726} of FTIR spectra as a function of concentration. (PEOX concentration and PAA concentration are accounted with the repeating unit of the polymer. The ratio between PEOX concentration and PAA concentration kept at 1.0, while the concentration increased from 1.0 × 10^{-3} mol/L to 8.0 × 10^{-2} mol/L)

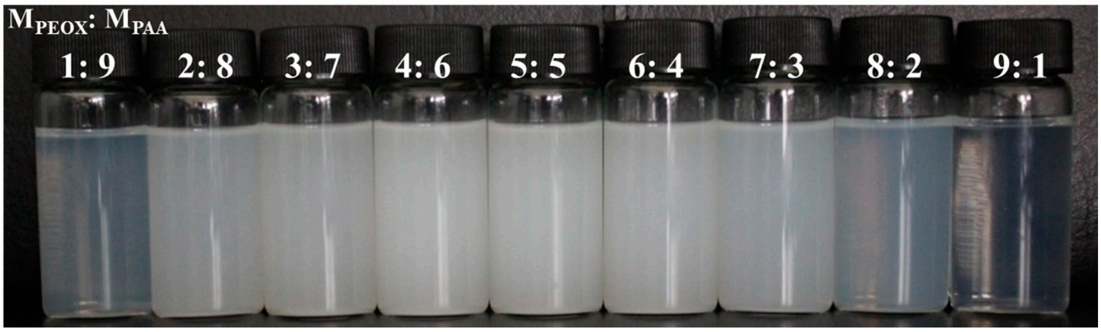

Figure S8. The photographs of the PEOX and PAA solutions mixed in different concentration ratio.

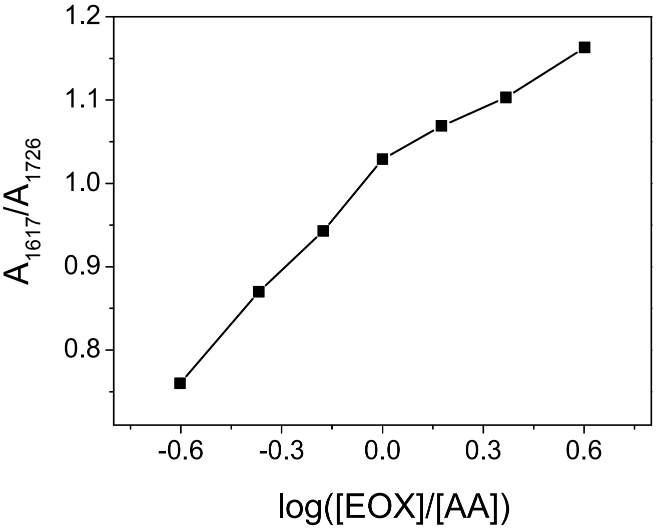

Figure S9. A_{1617}/A_{1726} of FTIR spectra as a function of log([EOX]/[AA]). (PEOX concentration and PAA concentration are accounted with the repeating unit of the polymer. Total concentration of PEOX and PAA kept constant, 1.0 × 10^{-2} mol/L)

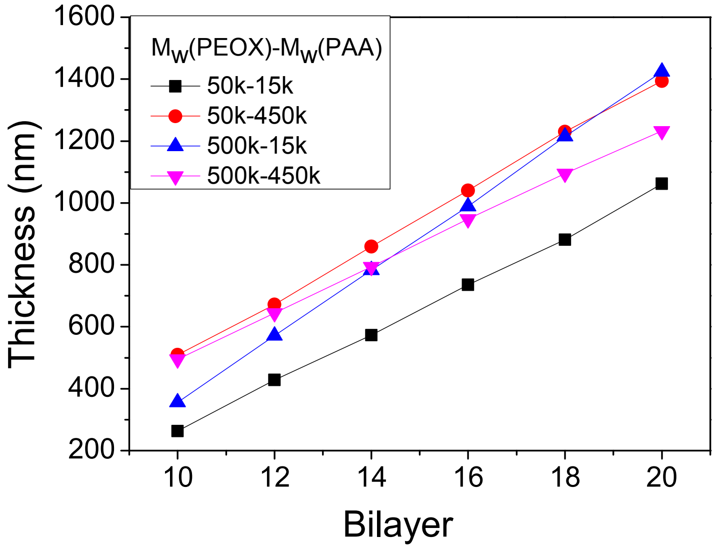

Figure S10. The thickness as a function of assembly cycles from 10 to 20. The films were prepared with different molecular weight of PEOX and PAA. (PEOX, [EOX]=1.0 × 10^{-2} mol/L; PAA, [AA]= 1.0 × 10^{-2} mol/L; pH, 2.0; assembling time, 4 min; rinsing time, 1 min; and room temperature)
